# Addressing inequities in maternal health through postpartum nurse home visiting: a mixed methods evaluation

**DOI:** 10.1186/s12884-026-08979-5

**Published:** 2026-03-27

**Authors:** Sarah C. Haynes, Sarah Hartman, Jennifer E. Phipps, Katherine Rominger, Jasmine Cuellar, Paige D. Gilliland, Gina Daleiden, Leigh Ann Simmons

**Affiliations:** 1 Department of Pediatrics, UC Davis Health, 2516 Stockton Blvd, Sacramento, CA 95817 USA; 2 UC Davis Center for Health and Technology, 4610 X Street, Sacramento, CA 95817 USA; 3 UC Davis Perinatal Origins of Disparities Center, Sacramento, United States; 4 First Five Yolo County, Davis, CA USA; 5 Betty Irene Moore School of Nursing at UC Davis, Sacramento, CA USA

**Keywords:** Postpartum nurse home visiting, Maternal health inequities, Low-income families, Social and structural determinants of health, Breastfeeding support, Hispanic/Latino maternal health, Postpartum healthcare access barriers

## Abstract

**Background:**

The First Five Yolo Welcome Baby (WB) program, initiated in 2021, is a nurse home visiting initiative funded by local and federal dollars. It aims to address postpartum health in low-income, uninsured, or Medi-Cal insured families in Yolo County, California. Amid persisting inequities in maternal health, especially in low-income and historically marginalized communities, WB was designed to overcome barriers to postpartum care access and provide essential support for social, emotional, and physical wellbeing for mothers and their families. The purpose of this study was to evaluate this program in terms of key outcomes, including participant and provider experience and perceptions.

**Methods:**

This mixed-methods study examined quantitative and qualitative data from 541 WB program participants between March 2022 and February 2024. Quantitative data included service utilization, demographic information, mental health screenings, and breastfeeding outcomes. Qualitative data, gathered through semi-structured interviews with 30 participants (15 English-speaking and 15 Spanish-speaking) and 15 providers explored participant and provider perceptions of program benefits and challenges. Thematic analysis was used to categorize qualitative data, and results were integrated with quantitative findings.

**Results:**

Most program participants identified as Hispanic/Latino (58%) and over half had a language preference other than English (48.8%), primarily Spanish. Quantitative results revealed high postpartum visit completion (74.5% for both two- and six-week visits) and a breastfeeding continuation rate of 75.2% at three months. Depression and anxiety screenings indicated substantial mental health needs, with 13.9% screening positive for depression and 19.4% screening positive for anxiety. Qualitative findings highlighted six themes, including the importance of access to social support and community resources, overcoming logistical barriers for low-income families, and facilitating healthcare continuity for mothers and infants. Participants emphasized the role of nurse home visitors in bridging gaps in healthcare access and providing emotional support, particularly for families with a language preference other than English.

**Conclusions:**

This study underscores the critical role of postpartum nurse home visiting programs in addressing social and structural determinants of health and reducing inequities in maternal health outcomes. By connecting low-income families with healthcare and community resources, WB has shown promise in promoting maternal and infant health. Further research on similar programs in diverse and larger populations could enhance our understanding of their long-term impact on health equity.

**Supplementary Information:**

The online version contains supplementary material available at 10.1186/s12884-026-08979-5.

## Background

Compared to other high-income countries, the U.S. has alarmingly high rates of maternal morbidity and mortality [1]. In particular, women with low socioeconomic status face significant disparities in the incidence of adverse pregnancy outcomes, such as preterm birth, preeclampsia, and gestational diabetes [2, 3], which can negatively impact their health through postpartum and beyond [3]. Notably, women with public insurance are more than twice as likely to die from pregnancy-related complications compared to those with private insurance [4]. 

The postpartum period is critical for improving maternal health, especially among vulnerable populations [5–7]. Most pregnancy-related deaths occur in the first few weeks after birth [8]. Comprehensive postpartum visits (PPV) are essential for assessing biopsychosocial wellbeing, managing chronic diseases, and addressing issues that are common following childbirth, including mental health and breastfeeding support [6, 9]. However, adherence to American College of Obstetrics and Gynecology (ACOG) recommendations for postpartum follow-up is exceptionally low, especially among low-income women, with fewer than 60% of Medicaid recipients attending their scheduled PPVs [6, 10–16]. Barriers such as competing priorities, lack of social support, transportation challenges, and perceived discrimination significantly hinder access to necessary care [17–19]. Missed postpartum appointments can delay detection of risks for maternal morbidity and mortality, such as infection and preeclampsia, and result in missed opportunities for addressing social and structural determinants of health and providing breastfeeding support [6, 9, 20]. 

Postpartum nurse home visiting (HV) can mitigate these inequities by providing targeted support to mothers and their families. Nurse HV programs may identify early signs of postpartum depression, hypertension, and other common issues [21–23]. Postpartum nurse home visitors also can provide education and support for breastfeeding, screen and refer families to needed social services to address structural determinants of health, provide infant supplies, and educate families regarding safe sleep, infant feeding, and other infant care practices. While research has highlighted positive postpartum HV outcomes [22, 24–27], only 15% of Medicaid-insured, eligible families receive a home visit in the United States [28]. Rates are even lower in California. Recent data show that of the 405,000 California families who would have benefitted from HV, only 3.5% received HV services [29, 30]. These data demonstrate a need to better understand how postpartum HV might be disseminated more broadly to fill a key gap in postpartum care.

This mixed-methods study aimed to identify the perceptions of the benefits and value of postpartum HV and to explore shared priorities among participants, HV nurses, and community prenatal care providers around postpartum care and HV. We integrated quantitative programmatic data and qualitative interview data from a county-wide nurse HV program in Northern California to examine the benefits and value of postpartum nurse HV. Understanding these perspectives is crucial for developing effective, equitable, and sustainable postpartum HV programs that address disparities in maternal health outcomes.

## Methods

### The Welcome Baby: Road to Resilience program

The First Five Yolo’s Welcome Baby: Road to Resilience program (Welcome Baby) is a nurse HV program funded by local and federal dollars. Started in 2021, the goal of Welcome Baby was to proactively promote well-being in the postpartum period for low-income women and their families in the wake of the COVID-19 pandemic. Eligibility criteria included: (1) being within 2 weeks of hospital discharge from giving birth, (2) living in Yolo County, and (3) being uninsured or insured through California’s Medicaid program, Medi-Cal. Participants were recruited for Welcome Baby during prenatal visits, after delivery, before hospital discharge, or through self-referral. Participants were recruited in one of three ways: (1) self-referral through the website or through First Five Yolo, (2) referral from a healthcare provider (primary care, prenatal care, obstetric care), (3) in-person recruitment by a Welcome Baby nurse in the hospital after delivery.

Every Welcome Baby participant received a comprehensive nurse home visit that included a thorough physical exam of both the mother and newborn. Any concerning findings were discussed, and a plan was made to access their obstetric provider, pediatrician, or access the emergency department if medically necessary. The nurse also connected the dyad to community resources based on their need including WIC and Help Me Grow (HMG). If the mother or the baby did not have a current primary care provider, the nurse connected them to a medical home and addressed barriers to meeting well-baby and postpartum visit guidelines. The mothers also receive a thorough psychosocial screening, including depression, anxiety, and intimate partner violence. Those determined to be at-risk of perinatal mood disorders were connected to high-quality, culturally and linguistically appropriate treatment services within 30 days. Throughout the screening and clinical evaluation process, the nurse delivered a health literacy curriculum and provided education on feeding, infant sleep, newborn care and development, understanding when to call a doctor, lactation support, and postpartum mood. Nurses followed the home visit with phone calls as needed, and all families received a 3-month call to reconnect the family to available services and a medical home if those connections have been lost.

Based on the clinical findings and screenings, some families were offered secondary prevention services from a community health worker (CHW) trained through Healthy Families America. During the CHW visits, the Family Resilience and Opportunities for Growth (FROG) assessment was administered. Depending on the outcomes of the FROG assessment, additional screenings for mood disorders and social needs were completed. CHWs provided emotional and practical support to families, referred them to community services based on need, provided active case management, and followed up as needed to close referrals made to the family. Those with specific vulnerabilities are referred to expanded home visiting services available in Yolo County that most closely match the birthing dyad’s needs.

### Measures and data collection

Quantitative data included program data from Welcome Baby participants who delivered infants between March 1, 2022 and February 28, 2024 and came from four sources: (1) Data on services provided reported by the nurse home visitors during the home visit (referrals provided, supplies provided, breastfeeding support provided, results of depression screening); (2) Survey data collected from participants during the home visit (demographic information, insurance status, mental health screenings, breastfeeding at time of visit); (3) Survey data from a 3-month follow-up (breastfeeding confidence, breastfeeding at 3 months); and (4) Electronic health record data (attendance at postpartum visits and well-child visits). Surveys were administered verbally by nurses during the visit or if the family preferred, self-administered.). Follow-up surveys were administered verbally by phone by nurse home visitors or through an email link to an anonymous web survey.

Qualitative data were obtained through interviews with a sample of 30 former Welcome Baby participants and 15 providers who refer and provide care to Welcome Baby participants. Participants were recruited for interviews through text messages and mailed fliers. We enrolled the first 15 English-speaking and the first 15 Spanish-speaking participants who responded to the recruitment call. Each participant received a $50 gift card for participating. Interviews lasted approximately 45–60 min and were conducted by two staff researchers, one of whom was Spanish fluent, with experience in qualitative methods (KR, JC). Interviewers followed a semi-structured interview guide developed for this study using constructs from the Practical, Robust Implementation and Sustainability Model (PRISM) (Supplementary file A) [31]. PRISM is an implementation science framework that examines contextual factors in addition to Reach, Effectiveness, Adoption, Implementation, Maintenance (RE-AIM) outcomes [32]. Interviewers conducted analytic memos following interviews. Interviews were audio recorded and transcribed verbatim and Spanish transcripts were professionally translated.

### Data analysis and integration

We calculated descriptive statistics for all quantitative measures, including counts, percentages, means, and standard deviations.

To analyze qualitative data, we used thematic analysis [33, 34]. We first uploaded all transcripts into Dedoose qualitative software [35]. A team of 5 coders (SH, KR, JC, PG, JP) met to code the first two transcripts, generating and discussing initial codes. Researchers then independently conducted line-by-line coding using a constant comparative approach, whereby each new code was examined in relation to existing codes to refine the coding framework and develop overarching themes [36]. At least two coders coded all remaining transcripts. When a researcher generated a new code, they created and sent an email memo to the rest of the team describing the new code with its definition and use.

Coders also completed analytic memos periodically during the coding process to further refine codes, reflect on initial impressions, and highlight possible themes. Excerpts were then organized by code into a word document and refined by the research team. This process was conducted separately for the participant interviews (*N* = 30) and provider interviews (*N* = 15). We conducted member checking by sending summaries of themes to participants for feedback.

Quantitative data and qualitative data from both participants and providers were integrated using an integrated results matrix. We identified consistent themes that overlapped in the analysis of participant and provider interviews. We also identified quantitative data that belonged to these themes. Draft results matrices were discussed and refined by the larger research team.

This research adhered to the Declaration of Helsinki and was approved by the UC Davis Institutional Review Board. The qualitative components of this study are reported following the Consolidated Criteria for Reporting Qualitative Research (COREQ) guidelines.

## Results

### Quantitative results

A total of 541 participants received home visits during the first two years of the WB program (March 1, 2021-February 28, 2023). Table 1 shows the demographic characteristics of participants. Most families (58.0%) identified as Hispanic/Latino, 74 (13.7%) identified as Asian, 61 (11.3%) identified as non-Hispanic white, 22 (4.1%) identified as Black, and 18 (3.3%) identified as multiracial. Around half of participants (48.8%) had a language preference other than English. Of these, the most common language was Spanish (76.2%), followed by Farsi/Hindi/Punjabi/Urdu (11.5%), and Russian/Ukrainian (4.6%). Most women in the program (79.1%) had not previously given birth and were insured under Medi-Cal, California’s Medicaid program (82.4%). Seventy-five women (13.9%) screened positive for depression and 109 (19.4%) screened positive for anxiety.

**Table 1:** Demographic characteristics of participants

	*N (%)*
Race/Ethnicity	
Hispanic/Latino	314 (58.0)
Asian	74 (13.7)
White	61 (11.3)
Black	22 (4.1)
Multiracial	18 (3.3)
Alaska Native/American Native	5 (0.9)
Native Hawaiian/Pacific Islander	2 (0.4)
Unknown	45 (8.3)
Primary Language	
English	273 (50.5)
Spanish	198 (36.6)
Farsi/Hindi/Punjabi/Urdu	30 (5.6)
Russian/Ukrainian	12 (2.2)
Mandarin/Korean	4 (0.7)
Other	16 (3.0)
Unknown	8 (1.5)
Other Children	
0	428 (79.1)
1	61 (11.3)
2	8 (1.5)
> 2	0.2 (0.2)
Unknown	43 (8.0)
Insurance Status	
Medicaid	446 (82.4)
Uninsured	84 (15.5)
Other or unknown	11 (2.0)
Depression Screen*	
Positive	75 (13.9)
Negative	457 (84.5)
Unknown	9 (1.7)
Anxiety Screen*	
Positive	105 (19.4)
Negative	436 (80.6)

*Positive screens are defined as those with a score of greater than 0 on the PHQ-9 (for depression) or the GAD-7 (anxiety)

Table 2 shows a summary of key program outcomes. Nearly all families (93.9%) received at least one referral to a health or social service. More than half of women received referrals for child development services (66.0%) and social support services (54.7%). Many also received referrals for medical or dental services (47.3%), food and basic needs (39.6%), or crisis or emergency services (29.8%). WB participants had high rates of postpartum visit completion (89.2% for two-week visit, 81.6% for 6-week visit, and 74.5% for both visits) as well as high rates of breastfeeding at three months (75.2% giving at least some breastmilk at 3 months). 87.7% of participants reported feeling more confident in breastfeeding after the home visit. Participants also had high rates of well-child visit completion (97.8% for newborn visit, 87.4% for one-month visit, 99.3% for two-month visit).

**Table 2:** Summary of program outcomes

	*N* (%)
Referrals Received (*N* = 541)	
Medical/Dental Services	256 (47.3)
Child Development	357 (66.0)
Social Support	296 (54.7)
Food/Basic Needs	214 (39.6)
Crisis/Emergency/Safety	161 (29.8)
Postpartum Visits (*N* = 369)	
Completed 2-week visit	329 (89.2)
Completed 6-week visit	301 (81.6)
Completed both visits	275 (74.5)
Well-Child Visits (*N* = 278)	
Completed newborn visit	272 (97.8)
Completed one-month visit	243 (87.4)
Completed two-month visit	276 (99.3)
Up to date on 6 month vaccines (*N* = 244)	105 (43.0)
Breastfeeding at 3 months (*N* = 371)	
Exclusive breastfeeding	167 (45.0)
Some breastfeeding	112 (30.2)
No breastfeeding	92 (24.8)
Confidence in breastfeeding (N=349)	
Much more confident	219 (62.8)
A little more confident	87 (24.9)
Confidence stayed the same	43 (12.3)

### Qualitative results

We identified themes within six major categories across participant and provider interviews. Categories included: (1) Serving low-income families, (2) Improving postpartum mental health, (3) Promoting breastfeeding, (4) Improving infant health, (5) Improving maternal health, and (6) Serving families with a language preference other than English (LOE). Table 3 shows the integrated results matrix summarizing the quantitative and qualitative findings within these categories and the overarching meta-inferences.

**Table 3:** Integrated results matrix showing qualitative and quantitative findings and meta-inferences

Category	Quantitative findings	Qualitative: Participant perspective	Qualitative: Provider perspective	Meta-inferences
Overcoming barriers for low-income families	All participants were either uninsured or enrolled in California’s Medicaid program. Nearly all participants (90.5%) received at least one referral to a community health center or community-based organization. Most participants received supplies during their home visits.	HV can help overcome barriers like transportation that would prevent families from receiving medical care or getting needed supplies.	Providers would like to help provide additional needed services to their low-income patients but this is often outside of their scope of practice and knowledge. Nurse HV is an effective way to connect to other resources available to low income families.	Postpartum nurse HV presents a unique opportunity to meet the needs of low-income mothers by eliminating barriers to access for medical, social, and other community resources. Low-income participants successfully completed visits and connected to community resources.
Improving postpartum mental health	Among participants who were screened, 14.8% screened positive for depression and 22.2% screened positive for anxiety. Nurse home visitors provided referrals to behavioral health services as needed.	HV reduces anxiety around the baby’s health and increases confidence in caring for baby. HV also provides a sense of emotional support to participants.	The program can fill a crucial gap in recognizing early signs of postpartum depression and anxiety and referring to treatment.	Postpartum nurse HV provides a way to screen and provide resources for postpartum depression and anxiety, and provides emotional support to new mothers often unavailable in healthcare settings.
Promoting breastfeeding	Two-thirds of participants (67.1%) reported exclusive breastfeeding at the time of the home visit. Most participants reported that they felt much more confident (56.6%) or a little more confident (25.3%) about breastfeeding after the *Welcome Baby* home visit	Participants perceive breastfeeding support as a main benefit of the program. Many participants described that the nurse home visitor helped to address breastfeeding challenges and provide encouragement.	Providers often feel they are not able to give postpartum women the breastfeeding support that they need; the program helps to fill that gap, which contributes to better health outcomes for mothers and infants.	Postpartum nurse HV is an effective way to provide breastfeeding support and can improve breastfeeding confidence and duration.
Improving infant health	86.7% of participants completed their one-month visit and 99.4% of participants completed their two-month visit.	HV can identify urgent medical needs of babies and help them get timely care.	Seeing infants in their home environment offers a valuable opportunity for education around safe infant sleep and feeding practices.	Postpartum nurse HV can improve infant health through promotion of infant safety and identification of potential medical or social problems.
Improving maternal health	Nearly all *Welcome Baby* participants (97.8%) completed at least one postpartum visit, with nearly two-thirds of participants completing two post-partum visits.	HV can ensure that mothers are healing and help identify problems and make appointments as needed.	Seeing patients in their homes and having a better understanding of the person can help to build trust with the medical system and facilitate early identification of warning signs.	Postpartum nurse HV may be especially helpful for identifying postpartum hypertensive disorders, which are the leading cause of maternal mortality in women of color.
Serving families with a language preference other than English	More than half of women (59.5%) identified as Hispanic/Latina and 51.3% had a language preference other than English.	Bilingual nurse home visitors could improve the experience for patients and families.	Finding ways to provide more support for LOE patients and families is important to providers.	Many women with a Spanish language preference successfully participated in the program, but having bilingual nurse home visitors could improve the experience.

#### Category 1: Overcoming barriers for low-income families

Home visiting helped to overcome barriers, such as transportation and childcare, that would prevent low-income families from receiving medical care or getting the supplies they need.

“I only have one car and my husband takes it to work, so I’m left without transportation. I like the Welcome Baby program a lot more because the nurse came to my home.” -Participant.

*“They all asked me if I needed anything*,* if I needed any help*,* resource on anything to help because at the time*,* I was going through a little bit of a bad situation. They offered to help me [apply for a program through the utility company] to lower the payment… And they helped out with diapers*,* they helped out with a baby swing…I wasn’t working at the time*,* and they asked me if I needed anything for the baby… I was very grateful for that.” -Participant*.

Providers also reported that the connection to community resources was a valuable part of the program. Qualitative data supported quantitative data demonstrating that low-income families were successfully reached through the HV program, and nearly all participants received infant supplies as well as referrals to services in the community.

#### Category 2: Improving postpartum mental health

HV reduced the stress that many parents experienced when bringing home a new baby. Participants described how having the nurse visit their home helped them to feel more confident in their ability to take care of the baby and to address their concerns and worries about the baby’s health. For participants who were concerned about the baby’s weight, nurses were able to weigh the baby, assess feeding needs, and answer parents’ questions. Some parents who had general anxiety over the baby’s wellbeing were comforted by having the nurse examine the baby in the home and provide reassurance.

*“It put my mind at ease*,* so yeah. Just having her respond to me*,* it put my mind at ease*,* and I wasn’t—I get really bad anxiety*,* and I worry a lot. So I’m like*,* ‘Oh*,* my God*,* do I need to go to the ER? Should I just go in as a walk-in?’ All those things play in my mind like something’s wrong. Her responding back to me just made me feel better all around*,* and it’s just like*,* ‘Okay*,* I feel okay.’” -Participant*.

HV provided emotional support to participants and families during the postpartum period. Participants stated that their relationship with the nurse home visitor made them feel connected and less isolated. Both participants and providers noted the need for ongoing support past the initial postpartum period.

*“I think*,* for me*,* just having*,* a woman come and*,* like*,* kind of*,* be and just be present with me was really what I needed*,* I think.” -Participant*.

*All that support [Welcome Baby] was giving me…That was the best thing. You know*,* that it’s not only important just knowing about the baby or how to take care of the baby…it’s the mom and the son. That’s the best thing. They did care about me*,* even though I had him…”- Participant*.

Providers also identified the value of the program connecting their patients to mental and behavioral health services, which they recognized as a gap in care. Because patients often do not come back for a visit until several months postpartum, providers perceived HV as an effective way to screen patients early for postpartum depression and anxiety and intervene early when necessary. This supports quantitative data showing that all participants received screening for depression and were referred to behavioral health services in the case of a positive screen.

*“[With Welcome Baby] we’re able to get moms connected to behavioral health and mental health services that they need*,* that they wouldn’t have necessarily even been screened for*,* for another six weeks. And that just goes on to help the mom and the baby*,* and the whole family*,* to get them set up with the best possible chances they have for positive outcomes for both of them.” - Provider*.

#### Category 3: Promoting breastfeeding

Both participants and providers perceived breastfeeding support as a main benefit of the program. Many participants expressed that they registered for the program because they were seeking support with breastfeeding.

*“I felt like the nurse was really supportive and she helped me. She walked me through with the breastfeeding part*,* so she encouraged me because I was to the point where I was like*,* well*,* I’m not producing enough*,* so she just motivated me. And luckily*,* I breastfed till like seven months*,* and I felt way more supported when she came over because I didn’t have that support with my other kids. And she just kept motivating me. And she was giving me more tips and on what to do and how to do it*,* and not to stress myself out*,* because stressing out makes you stop producing milk. I felt really motivated by the nurses.” - Participant*.

*“And then some doctors do ask how the mothers are doing and everything*,* but they’ll say*,* “Well*,* I’ll give you some resources. I’ll give you some pamphlets*,*” and everything*,* but no. The nurse*,* like she really helped you. She got a one-on-one*,* like trying to breastfeed*,* latching the baby on*,* and everything.” - Participant*.

One participant identified that she felt pressure from the nurses to pursue breastfeeding even though she had decided to formula feed.

*“I know for me particularly*,* I feel like just maybe the state of California or like the hospitals*,* or even the person that came*,* there was a lot of recommendation that I try to breastfeed*,* which was fine*,* but I wasn’t able to breastfeed my first child. It was just something I wasn’t going to do this time with my other child. And I do feel like there was a little bit of pushing on that.” –Participant*.

Overall, the qualitative and quantitative data on breastfeeding support converge. Participants’ enthusiasm for the breastfeeding support they received is reflected in the survey data showing that participants reported higher confidence in breastfeeding after the home visit and experienced higher rates of continued breastfeeding compared to overall rates in the county, state, and nation.

#### Category 4: Improving infant health

Participants expressed that the nurse home visitor identified issues that may have otherwise gone untreated and could have led to adverse consequences. Nurses were able to use their knowledge of community clinics to obtain clinic appointments right away for mothers who needed further evaluation, and in some cases, sent the families to the emergency room.

*And then she checked the jaundice*,* and then she thought it was a little bit*,* if I’m not wrong*,* I think it wasn’t lowering*,* or it was just*,* like*,* a number up*,* then when in the hospital*,* so she helped me to make a really fast appointment in the clinic.- Participant*.

Participants also reported that nurse home visitors countered misinformation about safe sleep practices and feeding. One participant described that the nurse home visitor helped her understand her baby’s hunger cues after discovering that the baby was not gaining sufficient weight. She was then able to quickly make an appointment to address this issue and helped her obtain the supplies she needed to begin pumping. Significantly, participants were also more likely to take their infants to scheduled well-child visits in the clinic, reflected in the quantitative data.

#### Category 5: Improving maternal health

Nurse home visitors were able to identify both medical issues of postpartum mothers and potential social and structural determinants of health that could be addressed.

*“Well*,* I had a C-section with him*,* an emergency C-section*,* and I wanted to take care of myself. And with the baby*,* my only concern was if I was feeding him correctly and if he was being satisfied with the milk that I was giving him…she even asked me if I could give her permission to take a look of the C-section that I have. And I told her*,* yeah. And then she told me that I was healing good.” - Participant*.

*“She was not just there for the baby*,* she was there for me to help with what I needed…I was just having some discomfort and pain that I didn’t know where it was coming from. But she let me know when to call or when to be concerned. But it just helped me not stress out… She was there to really answer all my questions.” -Participant*.

Providers reported several instances of home visitors identifying postpartum pre-eclampsia before the mother was seen in clinic for a postpartum visit. Providers view this as a major benefit of the program, potentially saving women’s lives.

*“ [Welcome Baby] did a home visit*,* and they triaged back with us because they checked—the nurse checked the blood pressures*,* and then they communicated right back to us they were getting some high blood pressures when the mom was at home when they did the post-partum visit. So they triaged with us. For us to be able to see patients sooner than where she was on the usual two weeks.” - Provider*.

*“With the moms*,* a lot of times if it’s a normal delivery*,* they’re not following up with their provider for up to six weeks afterwards. And the baby’s getting followed up on*,* but that appointment doesn’t really include much follow up on the mom. So there’s been a few instances where the mother had a history of slight*,* elevated blood pressure*,* but then when I go in the home*,* it’s super elevated. And we’ve caught a few cases of postpartum preeclampsia*,* which were pretty severe*,* and the moms had to end up being hospitalized…” - Provider*.

Quantitative data also supported maternal health benefits for participants of the program, demonstrated by high rates of PPV completion.

#### Category 6: Serving patients with a language preference other than English

More than one-third of participants reported that Spanish was their primary language. Participants reported that having bilingual nurse home visitors could improve the experience for patients and families with a language preference other than English. Although interpreters were available by telephone during the visits, some participants who used this service reported that communication was more difficult, and they would have preferred connecting with a nurse who also spoke Spanish. Participants with Spanish-speaking family also mentioned that bilingual nurse home visitors would have improved their experience of the visit.

*“Well*,* the only thing that I could say was a translator*,* because my husband only knows Spanish. So he was in part of the interview*,* too*,* so he was asking me*,* what was she saying? And I was translating to him. Yeah. So I can only say a translator for some parents that*,* you know*,* one knows Spanish—I mean*,* one knows English and one doesn’t.”- Participant*.

## Discussion

This mixed-methods study identified several key benefits perceived by both participants and providers around postpartum HV programs. Our findings affirm those of previous studies demonstrating the positive influence of HV programs on breastfeeding initiation and continuation, emotional support, and connections between service utilizers and providers [21–23, 37]. HV has the potential to make a significant impact on maternal and infant health by identifying potential life-threatening issues and ensuring timely receipt of medical care. Significantly, participants who received HV services were more likely to attend well-child and postpartum visits at their home clinic. It is possible that the HV program improved participant trust in the medical system and in the providers that referred them to a program that they found helpful and supportive. Previous studies have also suggested that home visits facilitate trust between patient and provider through positive associations with healthcare providers and healthcare delivery systems [37, 38]. Given the legacy of mistrust in the medical system by historically marginalized groups, building trust is essential for long-standing partnerships between providers and community members. The postpartum period represents an opportune time to build these relationships to promote and support health for the woman, infant, and family going forward.

This study suggests the important role of nurse home visitors being physically present in the home. In the intimate setting, nurse and mothers/families can have more personal conversations that better enable the identification of needs and ensure referral to resources in the community that can fulfill these needs, such as organizations that provide housing and food support, legal resources, and resources for those in violent or unsafe situations. Our findings indicate that most participants had a need for at least one of these services, underscoring the importance of continued support during the postpartum period, and the unique ability of HV to identify the family’s needs. This contrasts sharply with being seen at the clinic where the ability to address social, emotional, and material needs is limited due to lack of time and availability of providers.

Despite participants’ and providers’ positive perceptions of HV, our study also emphasized several important questions about sustainability and scalability of the HV program. Given the growing language and cultural diversity of the US population, being able to provide HV services in participants’ preferred language, as opposed to having a translator, can limit broader dissemination of HV programs. Important next steps to address this barrier include outreach programs that increase the number of bilingual nurses.

HV programs are also costly to provide at the outset. However, early identification of health problems and improved adherence to recommended maternal and infant well visits are critical for prevention of chronic disease. Studies that use a life course lens suggest that prevention is associated with long-term cost savings [39]. Future longitudinal studies of postpartum HV programs should be conducted to assess intermediate and long-term health and economic outcomes.

While Welcome Baby increased trust between participants and providers, medical mistrust in the US remains a consideration, especially in the recent political milieu. Vulnerable groups like immigrants, especially those without documentation, are at high risk for poor health outcomes and may decline participating in HV programs due to fears of being reported. Future educational and outreach programs should engage community constituents on the best ways to reach high-risk groups and provide HV services.

Our findings also offer insights into how postpartum HV programs such as Welcome Baby could be disseminated more broadly to address gaps in postpartum care. Dissemination efforts should emphasize the program’s ability to reach low-income, racially and linguistically diverse families- populations often underserved in the postpartum period. Key lessons include the importance of integrating HV programs within existing perinatal care infrastructure and leveraging trusted providers to facilitate referrals and initial engagement. Participants’ experiences also highlight the value of flexible, family-centered delivery models that can accommodate language preferences and logistical barriers through bilingual staff and hybrid or telehealth options. Finally, findings underscore the need to communicate the dual focus of HV on maternal and infant health, which participants identified as a distinguishing feature that fostered trust and engagement. These insights can inform dissemination strategies aimed at expanding access to postpartum HV and ensuring cultural and contextual fit across diverse communities.

Our study’s strengths include its mixed methods design, focus on a low-income population, and inclusion of English- and Spanish-speaking participants in both the quantitative and qualitative portions. However, several important limitations should be considered. First, we enrolled the first 30 respondents into our qualitative study (15 each for English and Spanish). These participants were eager to participate in a qualitative research study and may not reflect the entire population. Second, we excluded patients who spoke a language other than Spanish and English in our qualitative interviews; thus, our findings may not represent the perspectives of birthing women and families who come from other linguistic backgrounds. Third, because our study included quantitative data only from participants who had successfully participated in the Welcome Baby program and did not include any type of randomization or control population, our findings may be affected by selection bias. Finally, our study includes only birthing women living in a small county in Northern California and therefore may not be generalizable to women across the state or within the US.

## Conclusions

Despite its limitations, this study provides important insights into how postpartum nurse HV may be used to improve the health of low-income mothers and their infants. Our study highlights the dire need for support during the postpartum period and identifies potential mechanisms by which HV programs can fill important gaps. Postpartum HV programs may have the potential to reduce disparities in maternal and infant outcomes through early identification of symptoms, referrals to healthcare delivery systems, and increased social supports. As HV programs continue to be implemented and adapted to new populations, developing and testing implementation strategies will be important for identifying ways to optimize reach, equity, and sustainability.

## Supplementary Information

Supplementary Material 1

## Data Availability

We will not make raw data or transcripts available due to concerns about participant privacy. We will make qualitative code applications available upon request.

## Code availability

We will make Dedoose code applications available upon request.
