# Supplementary material for: Addressing inequities in maternal health through postpartum nurse home visiting: a mixed methods evaluation
Source: BMC Pregnancy Childbirth. 2026 Mar 27;26:493. doi: 10.1186/s12884-026-08979-5 (PMC13147564; doi:10.1186/s12884-026-08979-5)
Supplement: Supplementary file 1 — Supplementary Material 1 [file 12884_2026_8979_MOESM1_ESM.docx]

**Welcome Baby – Semi-structured interview guide for PROVIDERS**

|  | **Interview questions** |
| --- | --- |
| ***PRISM DOMAIN*** |  |
| **Intervention (Organizational perspective)** | - Tell me about the Welcome Baby Program at your clinic. - What are your overall thoughts about the program? - Do you have any previous experience with home visiting programs? How does this compare? - What are your thoughts on how the program is going? What does your team think? - How valuable is the intervention for your patients? - Is the intervention easy to deliver? Has it posed any burden on you/your team? |
| **Recipients (Organizational characteristics)** | - Is there overall support within your clinic for Welcome Baby? - Would you say that leadership at your clinic shares your goals with respect to caring for postpartum women (and specific to Welcome Baby?) |
| **Implementation & Sustainability Infrastructure** | - What would it take to make this a permanent program at your clinic? - Are there characteristics of your clinic that help to facilitate or to impede implementation of Welcome Baby? - What are your thoughts on expanding to other sites? |
| ***RE-AIM OUTCOME*** | |
| **EFFECTIVENESS** | - Is this program effective for improving maternal health? For improving infant health? |
| **ADOPTION** | - How was this program introduced to you/your clinic/other? - Why did you decide to adopt the program? - Are there others in your clinic or elsewhere that have chosen not to adopt Welcome Baby? |
| **IMPLEMENTATION** | - What is your role in this program? - Walk me through a typical patient interaction/visit for this program. - Tell me about a time you were unable to complete a clinic visit. - How does this program fit into your workflows? - What works best about this program? For you? For patients? For the clinic? - Have changes been made to program since first starting? - Is there anything about this program you would like to change? - What suggestions or tips would you give to someone wanting to implement this program in a different region? |
| **MAINTENANCE** | - Do you want this program to continue? Why or why not? - Do you intend to keep playing your role in the program? |
| **CLOSING** | - Is there anyone else you would recommend we speak with? - Any other thoughts you would like to share? |

**Welcome Baby – Semi-structured interview guide for PARTICIPANTS**

|  | **Interview questions** |
| --- | --- |
| ***PRISM DOMAIN*** |  |
| **Intervention (Patient perspective)** | - Tell me about your experience with the Welcome Baby Program - Why did you decide to participate? - Do you think the program is valuable? - Were there other similar options that you could have chosen? |
| **Recipients (Patient characteristics)** | - What were/are your priorities around postpartum? - What did you need the most during this period? Did the program meet those needs? |
| ***RE-AIM OUTCOME*** | |
| **EFFECTIVENESS** | - Did this program change your health or your baby’s health? - What are the benefits of this program? - What was your overall experience? |
| **IMPLEMENTATION** | - What is your role in this program? - Tell me about a time you were unable to complete a visit. - What works best about this program? For you and your family? - Is there anything about this program you would like to change? |
| **CLOSING** | - Is there anyone else you would recommend we speak with? - Any other thoughts you would like to share? |
